# Cost-effectiveness analysis of stroke volume variation guided perioperative hemodynamic optimization

**DOI:** 10.1186/cc12129

**Published:** 2013-03-19

**Authors:** J Benes, J Zatloukal, A Simanova, I Chytra, E Kasal

**Affiliations:** 1 The Medical Faculty and Hospital in Plzen - Charles University Prague, Plzen, Czech Republic

## Introduction

Perioperative goal-directed therapy (pGDT) can substantially improve the outcome of high-risk surgical patients [1]. But the approach needs an initial investment and increases the staff workload. Economic factors might participate in the weak adherence to the pGDT concept. Some model studies support pGDT cost-effectiveness, but real economic data based on a recent clinical trial are lacking. We performed an economic analysis of hemodynamic optimization using the stroke volume variation trial [2] in order to elucidate this issue.

## Methods

The hospital care invoices of all 120 patients included in the trial were retrospectively extracted. Due to the nature of the data we have adopted the healthcare payer's perspective. We performed a comparison of induced costs between the Vigileo (*n = *60) and Control (*n = *60) groups and constructed a cost tree using the study group and complications occurrence as distributive parameters. The incremental cost-effectiveness ratio per complication avoided was calculated and, finally, different reimbursing categories were assessed as potential cost drivers.

## Results

A decreased rate (18 vs. 35 patients) and number of complications (34 vs. 78) were observed in the original trials Vigileo group. The mean cost of intervened patient was lower (€2,877 ± 2,336 vs. €3,371 ± 3,238; *P *= 0.38). According to the cost-tree analysis, patients with complications (*n = *53; 44%) consumed significantly more resources (€235,623; 63%). A gain of €634 per avoided complications confirms that the lower complications rate was the most important cost driver. Both the clinical care for patients costs (€505 vs. 912; *P *= 0.04) and ward stay costs (€244 vs. 402; *P *= 0.03) were decreased by the intervention. On the contrary, the intervention increased anaesthesia costs (€880 vs. 688; *P *= 0.001).

## Conclusion

Intraoperative fluid optimization with the use of stroke volume variation and the Vigileo/FloTrac system showed not only a substantial improvement of morbidity, but was also associated with an economic benefit. This observed benefit highly exceed the increased monitoring costs in our trial.
